# Association of Chorioamnionitis with Aberrant Neonatal Gut Colonization and Adverse Clinical Outcomes

**DOI:** 10.1371/journal.pone.0162734

**Published:** 2016-09-22

**Authors:** Kriti Puri, Diana H. Taft, Namasivayam Ambalavanan, Kurt R. Schibler, Ardythe L. Morrow, Suhas G. Kallapur

**Affiliations:** 1 Division of Neonatology and the Perinatal Institute, Cincinnati Children's Hospital Medical Center and the University of Cincinnati, Cincinnati, OH, United States of America; 2 Division of Neonatology, University of Alabama at Birmingham, Birmingham, AL, United States of America; University of Liverpool, UNITED KINGDOM

## Abstract

**Objective:**

Chorioamnionitis (inflammation of the placenta and fetal membranes) and abnormal gastrointestinal colonization have been associated with an increased risk of sepsis and death in preterm infants, but whether chorioamnionitis causes abnormal pioneering gastrointestinal colonization in infants is not known. We determined the relationship between chorioamnionitis, altered infant fecal microbiome indicating abnormal gastrointestinal colonization, and adverse outcomes.

**Study Design:**

Preterm infants ≤ 28 weeks at birth were enrolled from 3 level III NICUs in Cincinnati, Ohio and Birmingham, Alabama. Sequencing for 16S microbial gene was performed on stool samples in the first 3 weeks of life. Chorioamnionitis was diagnosed by placental histology. Late onset sepsis and death outcomes were analyzed in relation to fecal microbiota and chorioamnionitis with or without funisitis (inflammation of the umbilical cord).

**Results:**

Of the 106 enrolled infants, 48 infants had no chorioamnionitis, 32 infants had chorioamnionitis but no funisitis (AC), and 26 infants had chorioamnionitis with funisitis (ACF). The fecal samples from ACF infants collected by day of life 7 had higher relative abundance of family Mycoplasmataceae (phylum Tenericutes), genus Prevotella (phylum Bacteroidetes) and genus Sneathia (phylum Fusobacteria). Further, AC and ACF infants had higher incidence of late-onset sepsis/death as a combined outcome. Presence of specific clades in fecal samples, specifically, order Fusobacteria, genus Sneathia or family Mycoplasmataceae, were significantly associated with higher risk of sepsis or death.

**Conclusion:**

The results support the hypothesis that specific alterations in the pioneering infant gastrointestinal microbiota induced by chorioamnionitis predispose to neonatal sepsis or death.

## Introduction

Aberrant gastrointestinal colonization has been associated with an increased risk for postnatal sepsis and necrotizing enterocolitis (NEC) in the preterm infant [1–5]. Treatment with beneficial bacteria decreases the risk of NEC and mortality in preterm newborns [6–8]. On the contrary, prolonged use of antibiotics in preterm infants is associated with increased risk for NEC, death and sepsis [9, 10]. Therefore, understanding the origins of intestinal dysbiosis in the preterm infant is important. It is now known that infant gastrointestinal tract, long thought to be sterile at birth is colonized with microorganisms [11–13]. Therefore, factors in the intrauterine environment likely play an important role in the initial colonization of infant gastrointestinal tract with microorganisms.

Fetuses actively swallow amniotic fluid *in utero*. Recent studies have demonstrated that amniotic fluid is often not sterile, and the profile of microorganisms in the amniotic fluid is different during intrauterine infections [14, 15]. The maternal and fetal immune response to intrauterine infection/inflammation manifests as neutrophil infiltration of the chorioamnion (chorioamnionitis) and umbilical cord (funisitis) respectively [16]. Although chorioamnionitis is present in nearly all cases of funisitis, funisitis is present in only about 30–60% of cases of chorioamnionitis [17]. Exposure to chorioamnionitis increases the risk of preterm infants to adverse neonatal outcomes including early onset sepsis and NEC [18–22]. Despite chorioamnionitis being a common exposure, diagnosed histologically in about 50% of women delivering at <28weeks gestation [23], the effects of chorioamnionitis on gastrointestinal microbial colonization are not known. We hypothesized that exposure to chorioamnionitis and/or funisitis will be associated with aberrant gut microbiota and that this profile of aberrant gastrointestinal microbiota will correlate with adverse neonatal outcomes (sepsis, NEC, death) in preterm infants. To ensure generalizability of findings, we enrolled preterm infants from NICUs in two geographically different locations, since we previously reported that fecal microbiota in preterm infants differ by hospitals in which they are cared for [24].

## Methods

### Study Infants

A total of 106 study infants born between 2009 and 2012 at two level III NICUs located in Cincinnati, Ohio and 1 level III NICU in Birmingham, Alabama were included for the study. This study was nested within a larger novel infant biomarkers of necrotizing enterocolitis study cohort [25]. The incidence of chorioamnionitis and adverse neonatal outcomes is the highest in the extremely preterm infants. Therefore to meaningfully understand the association between chorioamnionitis, aberrant pioneering gut colonization, and adverse neonatal outcomes, the inclusion criteria were: (i) infants born at ≤28 weeks of gestation and alive free of NEC prior to day 7 of life, (ii) placenta and umbilical cord sections available for review, and (iii) microbial sequencing available from stool sample ≤7 days of life. All infants remained free of NEC and sepsis in the first postnatal week and had no identified congenital anomalies. A total of 106 subjects met the inclusion/exclusion criteria. Written consent was obtained from the mothers of the infants enrolled in the study. The Institutional Review Boards at the 3 participating hospitals approved the study. In this cohort, early empiric antibiotic use consisted of ampicillin and gentamicin, using standard dosing. Most infants received at least 2 days of antibiotics. The duration of early empiric antibiotic therapy was defined as the total number of continuous days of administration of antibiotics with sterile culture results. Sepsis was defined as positive detection of organism in blood culture. Sepsis due to coagulase negative staphylococci was diagnosed if two consecutive blood cultures grew the same organisms or if the clinician ordered a complete course of antibiotics (≥5d treatment). NEC was defined using modified Bell stage II or III criteria [26].

Chorioamnionitis (inflammation in the free fetal membranes or chorionic plate of the placenta) and funisitis (umbilical cord inflammation) were diagnosed based on histopathology slides. Pathology slides from each patient from the Cincinnati study site was reviewed in a blinded fashion by two of the authors (KP and SGK) and another independently reviewed by a board certified pathologist. In most cases there was a good agreement between these two independent reviews. In the few cases of disagreements, the final determination was based on more elaborate adjudication based on reviewing additional slides from the same patient. The slides from the Alabama study site were reviewed by a board certified pathologist at the site and the final interpretation was the same as that recorded in the patient medical record. Chorioamnionitis and funisitis were diagnosed and severity classified histologically based on Redline criteria [27]. Infants were divided into three groups for analysis, infants without chorioamnionitis (NC), infants with acute chorioamnionitis but no funisitis (AC) and infants with acute chorioamnionitis and funisitis (ACF).

### Fecal microbiome

Serial stool samples were collected from infants during the first 3 weeks of life on days of life 5, 8, 11, 14 and 21 (±2 days for each time point). Samples were collected from soiled diapers, immediately refrigerated in the NICU, transported to the laboratory where they remained in the refrigerator until processing with thioglycollate and storage at -80°C. Bacterial DNA was extracted from infant stool samples [24]. The V4 region of the 16S ribosomal RNA gene was sequenced using the Illumina MiSeq platform by the Broad Institute (Boston, Massachusetts) using production protocols established for the Human Microbiome Project. The resulting sequence data was then processed as described by Taft et al [24].

### Statistical Analyses

The operational taxonomical unit (OTU) table generated from the sequencing data was first rarefied to 2000 reads per sample before any analysis. Consistent with our previous studies in preterm infants, we rarefied samples to 2000 reads based on the entire Novel Biomarkers of Necrotizing Enterocolitis cohort (a total of 1316 samples, described in ‘Results’ section); this depth provided a stable estimate of microbial composition while maximizing the number of infants with a successfully sequenced sample in the first 7 days of life. Alpha (within-sample) diversity was analyzed using the vegan package in the statistical computing program R (https://www.r-project.org) to calculate the Simpson diversity index and the Chao 1 metric. To analyze beta (between-sample) diversity, we first generated ordination plots using non-metric multidimensional scaling based on the weighted and un-weighted UniFrac distance calculated by QIIME[28]. We then used Linear Discriminant Analysis Effect Size Estimator (LEfSe) [29] to screen for differences in specific taxa by chorioamnionitis or funisitis status in sample collected during each week of life.

We classified the study infants *a priori* into three groups—no chorioamnionitis (NC), acute chorioamnionitis with no funisitis (AC), and acute chorioamnionitis with funisitis (ACF). The rationale for categorizing funisitis separately is that funisitis indicates fetal inflammation with infiltration of inflammatory cells of fetal origin in the umbilical cord [16]. During chorioamnionitis, inflammatory cells of maternal origin infiltrate fetal membranes[16]. Furthermore, increases in cord blood cytokines are largely restricted to funisitis cases, and chorioamnionitis is more severe when funisitis is also present [30, 31]. We then used a 3-way comparison between the study groups NC, AC and ACF, setting comparison criteria as one against all using a p-value of 0.05, and a minimum linear discriminant analysis (LDA) score of 2.0. We then used the statistical package R and Fisher’s Exact test to test the association between the presence/absence of the clades identified by LEfSe with each study group.

Study groups were then examined in relation to risk of late-onset sepsis (LOS), NEC or death, and combinations of these outcomes. Clinical outcomes that were significantly different in incidence between the groups were then analyzed for presence/absence of clades associated with those study groups. To check for potential confounders (gestational age, antibiotic use etc.) of the association between the adverse outcome and clades of interest, we first tested for an association between potential confounders and the outcomes of interest (at p≤0.1). The factors analyzed for potential confounding effect were as follows—infant gender, infant race, delivery mode, infant birth length, duration of antibiotic exposure in the first 7 days of life, maternal preeclampsia, infant gestational age, and the DNA extraction protocol used with the stool sample. If a potential confounder was identified, we then tested for association (p<0.1) between that confounder and the clade of interest by Fisher’s Exact test for categorical variables and ANOVA for continuous variables. We then used R to build logistic regression models that adjusted for potentially confounding variables. We were limited to adjusting for a single variable at a time to meaningfully analyze the low number of LOS cases and deaths within our cohort.

## Results

### Demographic and maternal variables (Table 1)

As expected, we had no cases of funisitis without chorioamnionitis. The study groups (NC, n = 48), (AC, n = 32), and (ACF, n = 26) were similar with respect to maternal characteristics, gender distribution, race and ethnicity, birth weight, and city of hospitalization. As expected, the NC group had a higher rate of maternal diagnosis of pre-eclampsia, and Cesarean section deliveries.

**Table 1:** Demographic characteristics and delivery data for study infants and mothers.

			Controls (NC) n = 48	Acute Chorio with no Funisitis (AC) n = 32	Acute Chorio with Funisitis (ACF) n = 26	p-value
**MATERNAL CHARACTERISTICS**	**Age at Delivery**	Mean ± S.D. (years)	28 ± 6	25 ± 5	27 ± 6	0.310
	**Insurance**	Medicaid (%)	19 (40%)^$^	16 (50%)	11 (42%)	0.230
	**Gravidity**	Mean ± S.D.	2.8 ± 2.4	2.6 ± 1.8	2.7 ± 1.9	0.800
	**Preeclampsia (%)**		17 (35%)	3 (9%)	1 (4%)	0.001*
	**Medications**	Antenatal Steroids (%)	48 (100%)	30 (94%)	26 (100%)	0.150
		Antenatal antibiotics (%)	22 (45.8%)	23 (71.9%)	22 (84.6%)	0.002*
**DELIVERY DATA**	**Mode of Delivery**	Cesarean Section (%)	39 (81%)	16 (50%)	12 (46%)	0.002*
	**Gestation**	Mean ± S.D. (weeks)	27.7 (1.8)	26.7 (2.1)	26.6 (2.1)	0.023*
	**Rupture of Membranes**	PPROM>72hours (%)	**6 (13%)**	**3 (9%)**	**10 (38%)**	0.032*
	**Apgar Scores**	1 min score <7 (%)	33 (69%)	25 (81%)	19 (73%)	0.490
		5 min score<7 (%)	8 (17%)	7 (23%)^#^	7 (27%)	0.530
**INFANT CHARACTERISTICS**	**Birth Weight**	Mean ± S.D. (grams)	1019 ± 275	1023 ± 231	933 ± 262	0.220
	**Gender**	Male (%)	18 (38%)	20 (62%)	13 (50%)	0.086
	**Ethnicity**	Hispanic (%)	2 (4%)	1 (3%)	0 (0%)	0.790
	**Race**	White (%)	37 (77%)	23 (72%)	15 (58%)	
		Black (%)	11 (23%)	8 (25%)	11 (42%)	0.180
		Other (%)	0 (0%)	0 (0%)	0 (0%)	
	**Days on antibiotics in first week of life**	Mean ± S.D. (days)	2.8 ± 1.9	3.5 ± 2.0	4.8 ± 2.1	0.000*

NC–no chorioamnionitis, AC–acute chorioamnionitis with no funisitis, ACF–acute chorioamnionitis with funisitis

* p <0.05

$ Mothers of 2 infants with ACF did not have insurance

# One AC infant did not have Apgar scores reported

### Fecal microbiome analysis

Since infant fecal microbial profile changes significantly in the first few weeks of life [12], we analyzed the association of chorioamnionitis or funisits with fecal microbiome separately in each week of postnatal life. In the full cohort, sequencing depth ranged from 594 reads to 321,900 reads. By selecting a rarefaction of 2000, we excluded 4 infants from this study who would otherwise have been eligible for inclusion. We noticed that sequencing depth was significantly associated with infant day of life at time of sample collection, with the lowest sequencing depth occurring in the earliest samples (when tested by ANOVA based on target day of life of collection, p = 0.002).

#### First Week of Life

**Fig 1:**
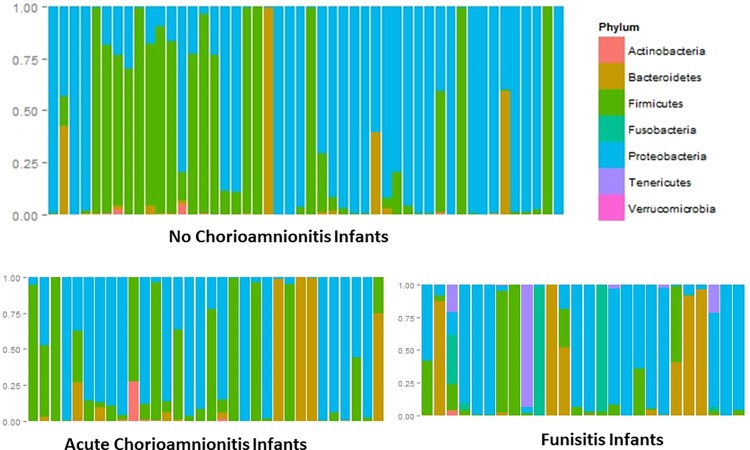
Barchart showing the relative abundance of bacteria in feces at the phylum level during week 1 of life. Each bar represents one infant. (A) No chorioamnionitis (NC) (B) Acute chorioamnionitis, no funisitis (AC), (C) Acute chorioamnionitis with funisitis (ACF). Infants exposed to funisitis have visibly more Tenericutes (purple) and Fusobacteria (blue green) than either of the other two groups.

**Fig 2:**
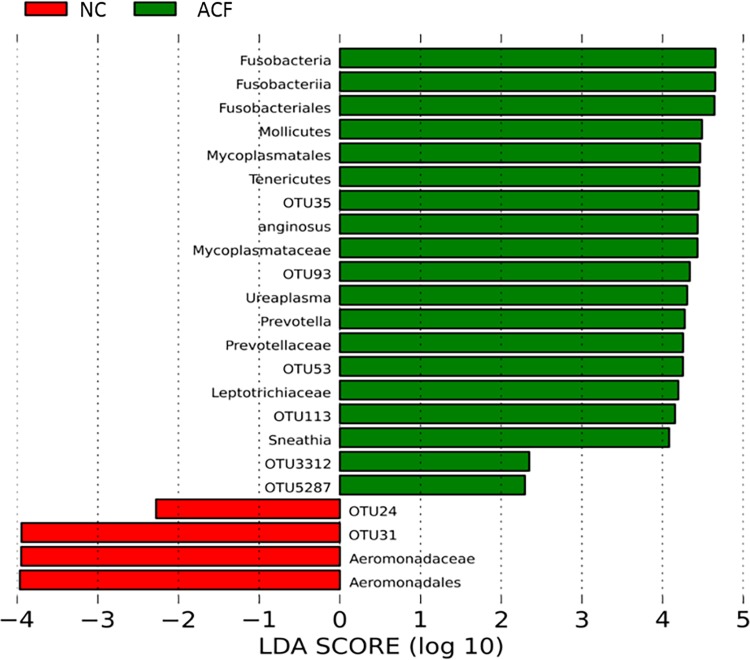
Differences in intestinal colonization in groups during the first week of life Red indicates taxa enriched in the no chorioamnionitis (NC) group. Green indicates taxa enriched in the acute chorioamnionitis with funisitis group (ACF). ACF infants had significantly higher levels of Mycoplasma, Fusobacteria and Prevotella. NC control infants had higher levels of Aeromonads.

Depiction of various phyla represented in the feces revealed that the infants exposed to funisitis (ACF) had visibly more Tenericutes (purple) and Fusobacteria (turquoise) than either of the other two groups, while the no chorioamnionitis group (NC) had more Firmicutes (green) (Fig 1). There was no significant difference in alpha (within sample) diversity across the three study groups. Principal component analysis did not reveal clusters on the ordinations based on either the weighted or the un-weighted UniFrac matrices (data not shown), indicating that there was no community-wide shift in microbial composition. Using LEfSe to compare the intestinal microbiota of the infants, differentially enriched taxa were observed only between NC and ACF in a 3-way comparison between NC, AC, and ACF. In the ACF group, there was a higher relative abundance of microorganisms belonging to family Mycoplasmataceae and genus Ureaplasma and the differences persisted at the higher taxanomic levels (Fig 2). Similarly, fecal samples from the ACF group had more relative abundance of Sneathia at the genus and higher order taxa level, genus Prevotella, and species *Streptococcus anginosus* (higher order taxa were not enriched for Prevotella and *Streptococcus anginosus*) (Fig 2). On the other hand, fecal samples from NC were enriched in family Aeromonadaceae and order Aeromonadales. LEfSe also detected additional OTU level differences which are not reported here because of sequencing of the 16S rRNA gene does not consistently allow for classification of reads below the genus level. The heirarchial taxonomical nomenclature for these organisms is detailed in Table 2.

**Table 2:** Hierarchical taxonomical nomenclature for microorganisms differentially detected in samples^#^.

**Phylum**	Tenericutes	Fusobacteria	Bacteroidetes	Firmicutes	
**Class**	Mollicutes	Fusobacteria			
**Order**	Mycoplasmatales	Fusobacteriales			Aeromonadales
**Family**	Mycoplasmataceae	Leptotrichiaceae	Prevotellaceae		Aeromonadaceae
**Genus**	Ureaplasma	Sneathia	Prevotella	Streptococcus anginosus	

# as referenced in Fig 2.

To further explore the differences detected by LEfSe analyses, we analyzed differences in proportion of subjects with detection of either genus, family or order level of organisms (presence or absence of organism) across the different groups (Table 3). We confirmed the differences in genus Sneathia, family Mycoplasmataceae, and genus Prevotella in the feces when analyzing on the basis of presence or absence. There was borderline greater prevalence in the ACF group for order Fusobacteriales and genus Ureaplasma. The differences between NC and the combined (AC+ACF) group were the same as those found between NC and ACF and a dot plot of the differential distribution in taxa revealed that almost all the differences were contributed by ACF (data not shown). Thus, ACF was a significant determinant of aberrant fecal microbial colonization. Reassuringly, the higher relative abundance of Fusobacteria, Mycoplasma and Prevotella in the ACF group remained when each center was analyzed separately suggesting a generalizability of the findings.

**Table 3:** Detection of specific clades in fecal samples of study infants in the first week of life.

MICROBE	NO. (%) OF INFANTS WITH DETECTABLE LEVELS OF MICROBE ^#^			
	NC (N = 48)	AC (N = 32)	ACF (N = 26)	p-value
Order Fusobacteriales^a^	3 (6.2)	2 (6.2)	6 (23.1)	0.080
**Genus Sneathia**^**b**^	**0**	**0**	**3 (11.5)**	0.013*
**Family Mycoplasmataceae**^**c**^	**1 (2.1)**	**3 (9.4)**	**8 (30.8)**	0.001*
Genus Ureaplasma^d^	1 (2.1)	1 (3.1)	4 (15.4)	0.062
**Genus Prevotella**^**e**^	**1 (2.1)**	**3 (9.4)**	**8 (30.8)**	0.001*
**Family Aeromonadaceae**^**f**^	**11 (22.9)**	**4 (12.5)**	**0**	0.016*

#—detectable levels on LEfSe defined as at least two reads belonging to a particular taxa

*—p <0.05 by Fisher’s exact test using 3x2 table (comparing presence/absence of negative outcome across all three chorioamnionitis conditions (NC, AC, and ACF)

NC–no chorioamnionitis, AC–acute chorioamnionitis with no funisitis, ACF–acute chorioamnionitis with funisitis. Taxanomic ranks, listed from highest to lowest used here, are phylum, class, order, family, and genus.

a–Phylum Fusobacteria, Class Fusobacteria. Phylum, class, and order had the same p-value

b–Phylum Fusobacteria, Class Fusobacteria, Order Fusobacteriales, Family Leptotrichiaceae. Family and genus had the same p-value

c–Phylum Tenericutes, Class Mollicutes, Order Mycoplasmatales. Phylum, class, order, and family had the same p-value

d—Phylum Tenericutes, Class Mollicutes, Order Mycoplasmatales, Family Mycoplasmataceae. Genus had a different p-value from all other taxanomic levels

e–Phylum Bacteroidetes, Class Bacteroidetes, Order Bacteroidales, Family Prevotellaceae. Family and genus had the same p-value

f–Phylum Proteobacteria, Class Gammaproteobacteria, Order Aeromonadales. Order and family had the same p-value

#### Second and Third Week of Life

**Fig 3:**
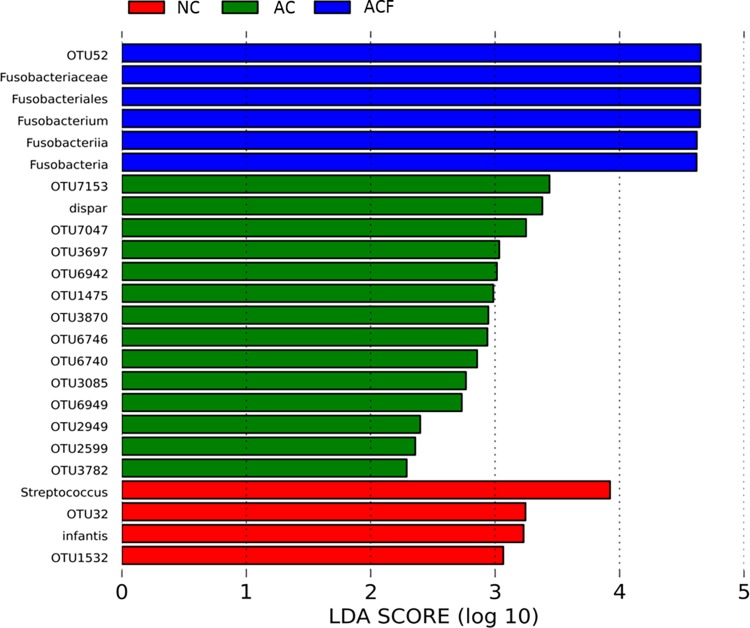
Differences in intestinal colonization by funisitis status during the second week of life. Red indicates taxa enriched in the group no chorioamnionitis (NC) group. Green indicates taxa enriched in the chorioamnionitis without funisitis group (AC). Blue indicates taxa enriched in the acute chorioamnionitis with funisitis group (ACF). ACF infants had higher levels of Fusobacteria and NC control infants had higher levels of Streptococcus.By the third week of life, the only difference between the NC, AC, and ACF groups above the OTU level was an enrichment of genus Clostridium (and species *Clostridium neonatale*) in the AC group (data not shown.) The persistence of the Fusobacteria signal from week 1 to week 2, and its absence in week 3 suggests that the detectable impact of chorioamnionitis on the fecal microbiome had vanished by week 3 of life.

Similar to results in the first week, fecal samples from the ACF group in the second week had higher levels of Fusobacteria compared to AC and NC groups, and the differences persisted at higher taxonomy (Fig 3). Consistent with these findings, compared to AC or NC group, fecal samples from ACF had higher numbers of subjects with Fusobacterium when analyzed as presence or absence of organism (p = 0.015). However, the differences seen in week 1 in presence of family Mycoplasmataceae, Genus Prevotella, or *Streptococcus anginosus* were no longer detected in week 2 samples. In week 2 samples, NC had higher levels of species *Streptococcus infantis* and genus Streptococcus compared to AC or ACF. The association between the presence/absence of species *Streptococcus infantis* and NC was also significant (p<0.001), and these were new associations compared to week 1. Compared to ACF and NC, AC infants had higher levels of species *Veillonella dispar*. All other differences were only at the OTU level and not considered clinically significant.

#### Association of chorioamnionitis, and aberrant microbiota with adverse clinical outcomes

Next, we examined for the association of chorioamnionitis with or without funisitis with adverse outcomes (Table 4). Of the 14 patients developing LOS or death, 12 were exposed to chorioamnionitis (AC + ACF), of whom 5 were in the ACF group. There was no difference in the incidence of LOS, NEC, or death between the groups (Table 4). However, when LOS and death were combined as a single outcome, compared to the NC group, infants with AC and ACF had a higher incidence of the LOS or death outcome. Nearly 50% of LOS was due to Coagulase negative Staphylococci (all of whom were classified as having sepsis based on a single positive blood culture followed by receiving a full course of antibiotics), with *Enterococcus faecalis* and Klebsiella accounting for 18% of sepsis cases each (Table 5).

**Table 4:** Incidence of adverse clinical outcomes among study infants.

CLINICAL OUTCOME	NC (N = 48)	AC (N = 32)	ACF (N = 26)	p-value
	No. (%)	No. (%)	No. (%)	
LOS	2 (4.2)	5 (15.6)	4 (15.4)	0.130
NEC	2 (4.2)	4 (12.5)	1 (3.8)	0.356
Death	1 (2.1)	2 (6.2)	2 (7.7)	0.514
**LOS or death**	**2 (4.2)**	**7 (21.9)**	**5 (19.2)**	0.033*
NEC or death	3 (6.2)	**5 (15.6)**	**3 (11.5)**	**0.360**
NEC, LOS, or death	4 (8.3)	8 (25)	5 (19.2)	0.12

NC–no chorioamnionitis, AC–acute chorioamnionitis with no funisitis, ACF–acute chorioamnionitis with funisitis, NEC–necrotizing enterocolitis; LOS–late-onset sepsis

*—p <0.05 by Fisher’s exact test, using 3x2 table (comparing presence/absence of negative outcome across all three chorioamnionitis conditions (NC, AC, and ACF)

**Table 5:** Characteristics of infants with adverse outcome sepsis or death.

Subject	Cohort	Gestational age at birth (weeks)	Age at Sepsis (days of life)	Organism Cultured from Blood	Death (age)
1	NC	27	13	*Enterococcus faecalis*	Yes (5weeks)
2	NC	24	8	Methicillin-resistant Staphylococcus aureus	No
3	AC	27	30	*Klebsiella pneumoniae*	No
4	AC	24	NA	NA	Yes (2weeks)
5	AC	24	9	Staphylococcus (coagulase negative)	No
6	AC	27	12	Staphylococcus (coagulase negative)	No
7	AC	24	NA	NA	Yes (11weeks)
8	AC	24	17	*Enterococcus faecalis*	No
9	AC	27	8	Staphylococcus (coagulase negative)	No
10	ACF	25	40	Klebsiella	No
11	ACF	24	NA	NA	Yes (4weeks)
12	ACF	25	12	Staphylococcus (coagulase negative)	No
13	ACF	24	8	Staphylococcus (coagulase negative)	No
14	ACF	23	11	*Escherichia coli*	Yes (2weeks)

NC–no chorioamnionitis, AC–acute chorioamnionitis with no funisitis, ACF–acute chorioamnionitis with funisitis; NA–not applicable

Since intraamniotic infection was associated with adverse clinical outcomes and aberrant fecal microbiota, we then tested the association between the fecal microbiota overrepresented in chorioamnionitis with or without funisitis and adverse clinical outcomes. Of the microbes associated with chorioamnionitis with or without funisitis reported in Table 3, only Family Mycoplasmataceae (p = 0.0009) and genus Sneathia (p = 0.045) emerged as significantly associated with the outcomes of LOS or death. Since colonization with Mycoplasmataceae and Sneathia were significantly associated with the adverse outcomes of LOS or death, we created a model to adjust for gestational age and antibiotic duration, to gain a quantitative sense of the association of the single factor defined as presence of Mycoplasmataceae or Sneathia (Table 6).

**Table 6:** Association of presence/absence of specific microbes with adverse clinical outcomes among study infants.

CLINICAL OUTCOME	Genus Sneathia+	Family Mycoplasmataceae+	Either microbe*	Either microbe	
				O.R. (95% CI)	p-value
**LOS**, n = 11, no. (%)	2 (18.2)	4 (36.4)	5 (45.4)	**8.4 (1.6, 41.2)**	**0.005**
NEC, n = 7, no. (%)	1 (14.3)	1 (14.3)	1 (14.3)	1.7 (0.03, 16.9)	0.51
Death, n = 5, no. (%)	0 (0)	2 (40.0)	2 (40.0)	6.8 (0.5, 66.3)	0.086
**LOS or death,** n = 14, no. (%)	2 (14.3)	5 (35.7)	6 (42.9)	**7.6 (1.7, 32.4)**	**0.004**
NEC or death, n = 11, no. (%)	1 (9.1)	3 (27.3)	3 (27.3)	3.8 (0.5, 20.2)	0.100
**NEC, LOS or death**, n = 17, no. (%)	2 (11.8)	5 (29.4)	6 (35.3)	**5.5 (1.3, 21.0)**	**0.010**
Controls, n = 89, no. (%)	1 (1.1)	7 (7.9)	8 (9.0)	reference	

LOS = late onset sepsis, NEC = necrotizing enterocolitis

+ Sneathia belongs to Order Fusobacteria and Phylum Fusobacteria. Microorganisms in family Mycoplasmataceae belong to Phylum Tenericutes.

*Either microbe refers to identification of either genus Sneathia or family Mycolasmataceae

Potential confounders (gestational age, antibiotic use etc.) were assessed (as detailed in ‘Methods’) and were considered for inclusion in the final model only if they were associated (p<0.1) with both the sepsis or death outcome AND colonization with either family Mycoplasmataceae or genus Sneathia. NICU site was initially considered as a potential confounder, but a stratified analysis suggested that differences observed at each site were similar. Given site similarity, we proceeded with a unified analysis for the two centers. Only four potential confounders were found significantly associated with risk of sepsis or death–number of days on antibiotics during the first week of life, infant gestational age at delivery, infant birth NICU, and infant birth length. Of these, only the number of days on antibiotics during the first week of life and infant gestational age at delivery emerged significant when included in the model. Final ORs were adjusted for infant gestational age only, as antibiotic use was not significant in the same models with infant gestational age. After adjustment, this combined presence/absence variable remained significantly associated with all three outcomes: LOS (OR = 7.2 [95% CI 1.6, 33.7]); LOS or death (OR 9.0 [95% CI 1.8, 45.2], p = 0.008); and NEC, LOS or death (OR 5.6 [95% CI 1.2, 25.5], p = 0.026).

## Discussion

In a multi-center study setting, we report a novel finding of the association of chorioamnionitis or funisitis with aberrant early gastrointestinal microbiota in extremely preterm infants. A conundrum in the field is which antenatal factors influence initial neonatal gastrointestinal colonization.[13] Our findings clearly demonstrate that chorioamnionitis, a relatively common exposure in preterm infants,[23] is one potential factor. The association of aberrant gastrointestinal colonization with chorioamnionitis or funisitis was only demonstrated in week 1, with a fading signal in week 2 that disappeared by week 3, lending biologic plausibility for a casual association. Interestingly, the microorganisms belonging to Genus Sneathia and/or family Mycoplasmataceae, overrepresented in chorioamnionitis or funisitis cases, were correlated with later development of sepsis or death. Chorioamnionitis or funisitis were also correlated with increased incidence of sepsis or death. Thus, our data suggest a mechanistic role for specific aberrations in early neonatal gut microbiome in the development of later sepsis or death in infants exposed to intra-amniotic infection.

What constitutes normal gastrointestinal flora in preterm infants is not known, since preterm infants usually have multiplicity of exposures (antibiotics, holding feeds, feeding tubes) that would be expected to alter normal microbial gut colonization. The number of sequence reads in the samples collected from our preterm infant cohort (594 to 321,900) is notably lower than that reported in adult cohorts, which we attribute to the lower microbial colonization reported for early postnatal life compared to later infancy [32], and potentially, to the prematurity of our study infants. Compared to week 2 samples, we found a distinct meconium flora in week 1 fecal samples in extremely preterm infants, similar to findings reported by Moles et al [12]. We extend the findings of Moles et al by demonstrating that the initial meconium flora varies based on exposure to chorioamnionitis or funisitis. Using a case control study design, Mai et al reported predominance of Firmicutes [2] and Madan et al showed a predominance of Proteobacteria and Firmicutes (Staphylococcus) in stool from preterm infants developing late-onset sepsis [33]. Here, we examined a more limited set of taxa in relation to LOS, and found the strongest correlation to later sepsis was with Genus Sneathia (Phylum Fusobacteria) and family Mycoplasmataceae (Phylum Tenericutes). The risk of sepsis associated with Sneathia and Mycoplasmataceae might be mediated by the in utero exposure to inflammation from chorioamnionitis, making detecting this signal dependent on the correct classification of chorioamnionitis exposure. Other potential explanations for the different results of microorganisms associated with sepsis between our study and those of others are different study design, focus on chorioamnionitis, and differences in patient population or care/feeding practices. Several studies have correlated changes in fecal microbiota with later development of NEC. While the inter-individual variation in microbiome is large in these studies, some common themes are low microbial diversity, increased abundance of Enterobacteriaceae, Proteobacteria and decreasing Firmicutes, and Bacteriodes in infants who went on to develop NEC [1, 3, 34, 35]. We did not find associations between microbiota and NEC in our cohort, possibly due to the relatively low incidence of the disease in our study. Although more studies are needed, collectively the studies demonstrate an association of changes in gastrointestinal microbiota with outcomes in preterm neonates, and the potential for a causative role.

At term gestation, initial fecal microbiome in infants delivered vaginally reflected their own mother's vaginal microbiota, dominated by Lactobacillus, Prevotella, or Sneathia spp., and those delivered by C-section harbored bacterial communities similar to those found on the skin surface of their mothers, dominated by Staphylococcus, Corynebacterium, and Proprionibacterium [36]. Our data in extremely preterm infants revealed an increased abundance of microbes belonging to the family Aeromonadaceae in infants not exposed to chorioamnionitis, and increased abundance of phylum Fusobacteria (Genus Sneathia) and family Mycoplasmataceae in infants with exposure to chorioamnionitis with funisitis. There were no clades specifically enriched in the chorioamnionitis without funisitis group. The differences in fecal microbiome between no chorioamnionitis subjects were more striking when funisitis was also present in addition to chorioamnionitis. The degree of inflammation was in general higher in funisitis cases. Recent reports demonstrate detection of amniotic fluid microorganisms (predominantly Ureaplasma, Mycoplasma, Sneathia and Fusobacterium species) much more frequently in cases with more severe intrauterine inflammation [37]. Furthermore, Ureaplasma (Family Mycoplasmataceae, Phylum Tenericutes) and Sneathia (Phylum Fusobacter) are the most common organisms isolated from the amniotic fluid [14], and increased abundance of Tenericutes in amniotic fluid [14], or Ureaplasma in vaginal microbiota is correlated with preterm labor [38, 39]. Taken together, the predominance of the same organisms in infant stool as those previously reported in amniotic fluid microbial cultures, lend weight to the hypothesis that organisms causing chorioamnionitis or intrauterine infection also colonize the preterm infant gut via fetal swallowing. However we did not have culture reports from the placenta, umbilical cord or amniotic fluid to conclusively confirm this hypothesis. Interestingly, placental microbiome is more closely related to the oral microbiome than to the vaginal microbiome [40]. Further, we recently reported in another cohort of preterm infants that women who experienced spontaneous preterm labor harbor placental microbiota that differed by severity of chorioamnionitis. Integrative metagenomic analysis revealed significant decreases in butyrate and riboflavin metabolites in cases of severe chorioamnionitis [41]. Since these metabolites are known to have anti-inflammatory properties [42, 43], the data provide a plausible explanation for the association of severity of chorioamnionitis, placental microbiota, and local metabolites. Thus, chorioamnionitis may determine which organisms are able to colonize the preterm gut. Further studies will be needed to interrogate interactions between microbial communities in different maternal and fetal/neonatal niches during health and disease.

A major source of discrepancy in the chorioamnionitis outcomes literature is the definition of chorioamnionitis. In a large Neonatal Network study (NICHD) study of preterm infants ≤28 weeks gestation, the incidence of chorioamnionitis diagnosed by clinicians was 18% vs. histologic diagnosis of 48% [44]. In recent studies using the histologic definition, chorioamnionitis was associated with increased incidence of early-onset sepsis but not LOS [18, 20, 21]. We also did not find a significant association between chorioamnionitis and LOS, but chorioamnionitis was associated with LOS or death as a combined outcome. In our study, LOS was caused mostly by Coagulase negative Staphylocci (45% cases), *E*. *faecalis* or Kleibsiella (18% cases each). Well-known risk factors for LOS include prematurity, indwelling catheters, and nosocomial transmission of infection in the intensive care setting [45]. In large Neonatal Research Network studies in the US, the incidence of LOS was 36–45% [18, 44], while the incidence of LOS in our smaller study was 12%. It should be noted that all the three centers in our study also participate in the Neonatal Research Network and therefore contributed data to the reports by Pappas et al and Stoll et al. Whether the recent decrease in the incidence of LOS due to quality improvement efforts in care practices [45], now bring out associations that were previously hidden is not known. Regardless, our finding of the association of histologic chorioamnionitis, aberrant neonatal gut colonization and LOS/death outcome in extremely preterm infants warrant further investigation at a time of decreasing LOS.

The intestinal microbiota could translocate across the intestine and cause sepsis, or alternatively in cases where the causative organism of sepsis is not detected in the intestine prior to the onset of sepsis, intestinal dysbiosis may have altered local and systemic immune function [2, 33]. Elegant studies in mice have clearly demonstrated the role for intestinal bacterial flora in modulating local intestinal immune function, systemic T-cell and neutrophil function, which in turn modulated the risk for experimental sepsis [46–49]. We recently reported that preterm infants with chorioamnionitis and funisitis, and fetal Rhesus macaques with experimental intrauterine inflammation have decreased function of the anti-inflammatory T-regulatory cell subset [50, 51]. Thus aberrant immune function may be a missing link between chorioamnionitis, intestinal dysbiosis, and increased predilection for LOS. Since the neonatal sepsis and death outcomes are relatively rare, our findings may be limited by the sample size and will need confirmation in a larger cohort.

Previously reported association between chorioamnionitis and NEC was restricted to funisitis cases [22], but we did not find such a correlation. Smaller numbers of subjects and overall low incidence of NEC probably explain the discrepancies. The large numbers of subjects in clinical retrospective studies are not practical for the mechanistic metagenomic study in this report and indeed ours is one of the larger ones for a metagenomic study. Our earlier work on gastrointestinal colonization and LOS failed to detect an association with either Sneathia or Mycoplasmataceae [52] likely due to the difference in time windows used for analysis, as our prior work included samples collected in the first and the second week even in the earliest time period examined. This highlights the critical importance of time as a variable in seeking to understand the role of the intestinal microbiota in early life, as the preterm infant gut microbiome evolves rapidly in the first weeks of life, and even a difference of few days in timing can obscure the ability to detect a potentially important signal.

Our study was limited by the small numbers of the infants in the study. Hence we did not have adequate power to assess for clinical variables confounding the picture in cases of chorioamnionitis and/or funisitis and antibiotic exposure, for example, the obstetrician’s clinical response to the diagnosis leading to initiation of antibiotics in the mother and hence exposure to antibiotics for the fetus. We cannot precisely quantify the contribution of altered preterm gut microbiome alone to the adverse outcomes, since the preterm low birth weight infant cohort is a higher risk group with multiple noxious exposures. However, after adjusting for possible confounders, the association of colonization by Mycoplasma or Sneathia do emerge as a significant association with adverse outcomes. This is a potentially novel association which is important in the light of the previous reports of these bacteria being cultured from amniotic fluid in cases of chorioamnionitis. Further studies would benefit from corresponding cultures from amniotic fluid and placental or cord tissue, to confirm the origin of the organisms we are finding enriched in the guts of preterm infants exposed to intrauterine infection. We also did not analyze type of feeding as a confounder for this study, as all the infants enrolled in Cincinnati received either maternal breastmilk or donor breastmilk exclusively for the first 14 days of life.

In summary, we report a predominant early gut microbial colonization with Sneathia and Mycoplasmatacea in extremely preterm subjects with chorioamnionitis and funisitis. These organisms in the week 1 fecal samples were associated with increased risk for LOS or death and the diagnosis of chorioamnionitis and funisitis were also associated with adverse outcomes. Consistency in neonatal preterm gut microbial patterns, and clinical findings across two geographically distinct centers adds more confidence to the results. These findings prompt further investigations in to microbial functional genomic and metabolic perturbations induced by antenatal infections and impact on host immunity and susceptibility to infections in preterm infants.

## Abbreviations

AC: Acute Chorioamnionitis with No Funisitis
ACF: Acute Chorioamnionitis with Funisitis
NC: No Chorioamnionitis
OTU: Operational Taxonomical Unit
NEC: Necrotizing Enterocolitis
LOS: Late-onset Sepsis
